# Specialist Resource Centres as Protective Microsystems: A Qualitative Comparative Case Study of Autistic Pupils’ Experiences in Mainstream Secondary Schools

**DOI:** 10.1177/13623613261457949

**Published:** 2026-07-02

**Authors:** Alice Boddy, Anna Cook

**Affiliations:** 1 University of Surrey, Guildford, UK

**Keywords:** autism, specialist resource provision, inclusion bases, qualitative research, ecological systems theory, school belonging, mental health, mainstream secondary schools, special educational needs, school inclusion, school anxiety

## Abstract

Autistic pupils in mainstream secondary schools face significant barriers to academic progress, social engagement, and mental wellbeing, with parents consistently reporting that their children’s autism-related needs are not being met. This qualitative comparative case study examined what shapes the educational experiences of autistic pupils in secondary schools with Specialist Resource Centres (SRCs) versus comparable mainstream settings without specialist provision. Using Bronfenbrenner’s ecological systems framework, the study explored how multiple layers of influence – ranging from classroom practices to societal attitudes – interact to support or undermine inclusion. Data were collected across seven secondary schools (five SRC schools, two mainstream schools without SRCs) in South-East England via interviews with pupils, parents, and staff. Findings revealed that SRCs functioned as protective microsystems, providing predictable environments, visible support, and trusted relationships that buffered pupils from sensory and social demands. Key protective features included consistent staffing, flexible policies, and normalised support-seeking. However, systemic challenges – including workforce shortages, inadequate mental health services, and resource limitations – constrained the impact of even the most effective school initiatives. The study underscores that effective inclusion requires coordinated support spanning immediate school environments to broader policy frameworks, with implications for admission criteria, staff training, and inter-agency collaboration.

**Lay Abstract:**

Many autistic young people struggle in secondary school, facing difficulties with academic work, making friends, and managing their mental health. Parents often report that schools don’t properly understand or support their children’s needs. This study explored what makes school better or worse for autistic pupils by comparing two different types of schools: those with specialist autism units (called Specialist Resource Centres) and regular mainstream schools without these units. We spoke to autistic pupils, their parents, and school staff across seven schools in South-East England to understand their experiences. We found that specialist autism units acted like “protective spaces” within schools, providing quieter spaces, clearer routines, and staff who understood autism. These units helped manage sensory demands and social pressures. Pupils could use these spaces when they felt stressed and gradually build confidence to spend more time in mainstream classes. However, we also discovered that broader problems in the education system – like staff shortages, lack of mental health support, and limited resources – affected all schools, regardless of whether they had specialist units. Many autistic pupils still experienced high levels of anxiety and felt they had to hide their autistic traits to fit in. Our findings suggest that while specialist autism units can be helpful, creating truly inclusive schools requires changes at all levels – from individual classrooms to government policy – to provide better support for autistic young people’s education and wellbeing.

## Introduction

Autistic children and young people represent a significant and increasing group within England’s schools, with an estimated 73% educated in mainstream settings (Department for Education [DfE], 2022; National Autistic Society [NAS], 2011). This mirrors international inclusion agendas and global commitments to educating all learners in mainstream contexts (UNESCO. 1994, 2009). However, parents repeatedly report that autism-related needs go unmet (NAS, 2021). This is reflected in educational and social outcomes: autistic pupils face elevated risks of bullying, exclusion, and mental health difficulties (Carrington et al., 2017; DfE, 2018; Brede et al., 2017; Goodall, 2018a; Humphrey & Symes, 2010; Humphrey & Lewis, 2008). These gaps persist despite long-standing policy commitments to inclusion, grounded in the “progressive removal of barriers to learning and participation” (DfE, 2015, p. 25) and decades of debate about the balance between mainstream and specialist provision (Florian, 2019; Warnock, 1979). Although numbers in specialist schools have risen (Norwich, 2019), policy frameworks continue to emphasise meaningful participation in the mainstream through tailored support, reasonable adjustments and access to specialist expertise, placing the onus on schools to accommodate diverse learners (Kurth & Keegan, 2014; Rix et al., 2009; Strogilos et al., 2021).

Barriers are especially pronounced in secondary schools, where social, organisational, and sensory demands intensify (Ashburner et al., 2010; Goodall, 2018b; Humphrey & Symes, 2011). Anxiety, depression, and emotional dysregulation are disproportionately common (Hebron & Humphrey, 2012; Ridgway et al., 2024; Atkinson et al., 2025), alongside difficulties in friendship formation and maintenance, heightened exposure to stigma and bullying, and the use of masking to avoid peer rejection – often with detrimental effects on wellbeing (Cook et al., 2018; Carrington et al., 2017; Cook et al., 2016; Goodall, 2018a; Halsall et al., 2021; Humphrey & Lewis, 2008). Conversely, predictable routines, quiet spaces, and respectful staff–pupil relationships can buffer risk and support belonging and learning (Goodall, 2018a; Hummerstone & Parsons, 2021). Understanding how placement type shapes these experiences is therefore critical.

The prevalence of Specialist Resource Centres (SRCs) – autism resource provisions embedded in mainstream schools – has expanded to bridge this policy–practice gap. Typically framed as hybrid models that combine targeted support with mainstream participation, SRCs occupy an integrative position (DfE, 2017; ESFA. 2020; Ravet, 2011). Comparable hybrid or “satellite/resource-class” models exist internationally, aiming to pair specialist expertise and environments with continued access to mainstream peers and curricula. However, a national review within the Delivering Better Value in Special Educational Needs and Disabilities (﻿SEND) programme in England identified low parental confidence in mainstream and substantial mismatch between pupils’ needs and placements: 66% of autistic children and young people were judged to be in the wrong provision; while 14.7% were deemed best suited to SRCs, only 2.9% were actually placed, and SRC places have not kept pace with demand (DfE, 2024). Existing research suggests higher parental satisfaction with autism-specific SRCs than with mainstream-only or generic special educational needs (SEN) resource bases, alongside perceived benefits such as calmer spaces, staff expertise, flexible timetabling and coordinated support (Bond & Hebron, 2016; Frederickson et al., 2010; Hebron & Bond, 2017; Lindsay et al., 2016; Strogilos & Ward, 2024). Yet much of this literature is small-scale, single-site or descriptive, underlining the need for comparative evaluation to guide provision and policy (DfE, 2022).

**Figure 1.**
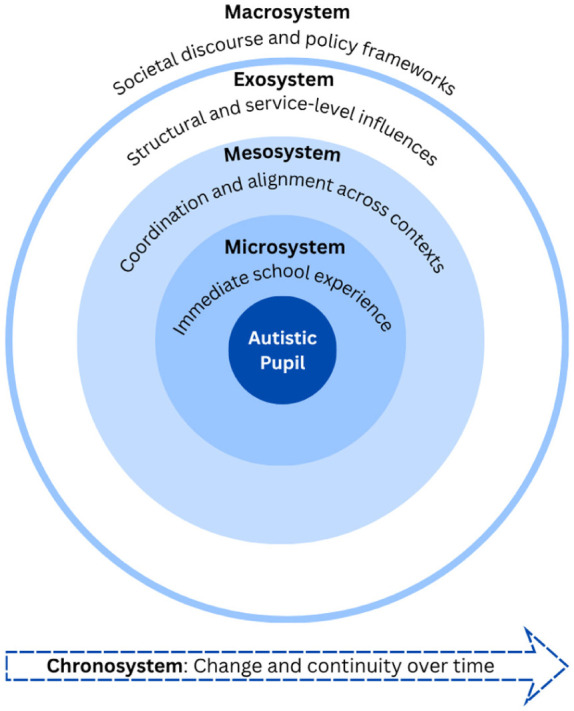
Nested ecological systems shaping autistic pupils’ school experiences (Bronfenbrenner’s ecological systems theory).

To examine how provision type interacts with wider influences, we adopted Bronfenbrenner’s ecological systems theory (Bronfenbrenner, 1979), which conceptualises pupils’ experiences as shaped by nested systems (Figure 1). Rather than viewing pupils’ experiences as the product of individual characteristics or single institutional factors, the ecological framework emphasises how multiple layers of context interact to influence wellbeing and participation. In school settings, these layers include immediate environments such as classrooms and peer relationships (microsystem), connections between settings such as home–school communication and professional collaboration (mesosystem), institutional and administrative structures including local authority processes and resource allocation (exosystem), broader societal discourse and policy frameworks shaping inclusion (macrosystem), and temporal dynamics reflecting change and continuity in experiences and support over time (chronosystem).

This framework is particularly relevant for examining SRCs, which operate as embedded environments within mainstream schools and therefore sit at the intersection of multiple ecological layers. Prior research on SRCs highlights the framework’s value for situating autistic pupils’ experiences as products of overlapping, interacting layers rather than a single factor (Bond & Hebron, 2016; Hebron & Bond, 2017). Using an ecological lens enabled us to examine not only the immediate supports available to pupils but also how organisational practices and wider system pressures shape the effectiveness of those supports across different school contexts. We also address educational change dynamics (Fullan, 2007), recognising that implementation depends on leadership ethos, staff learning, peer climate, and system incentives.

Against this backdrop, we present a multi-site comparative case study of seven secondary schools in South-East England – five with SRCs and two without – to ask: What shapes the educational experiences of autistic pupils in schools with and without SRCs? Extending previous research, we triangulate accounts from pupils, parents, and staff in a cross-case comparative analysis across five timepoints (Yin, 2009), enabling us to interrogate mechanisms that support or constrain pupils’ participation and wellbeing across ecological levels. By moving beyond surface description to examine how school structures, relationships, and system conditions interact, the study identifies when and how SRCs function as protective microsystems within mainstream schools, with implications for admissions, staff development, and inter-agency collaboration. [Complementary quantitative findings from the same evaluation are reported elsewhere (Cook & Boddy, 2026), providing additional context for the patterns identified here.]

## Method

### Design and Setting

We conducted a qualitative multi-site comparative case study across seven mainstream secondary schools in South-East England (five with SRCs; two non-SRC (NSRC)), spanning three local authorities, the administrative bodies responsible for administering education and social care services in those areas. Schools varied in socioeconomic profile, using eligibility for free school meals – a means-tested indicator of low household income in England – as an indicator (8.8%–33%; national average = 24.6%). Bronfenbrenner’s ecological framework informed the conceptual design of the study through an initial theory of change model that mapped nested influences within school environments. Data were gathered at five time points over 3 years (2021–2024) combining interviews, focus groups, and open-text survey responses (Table 1). While the multiple timepoints provide contextual breadth, capturing experiences across different stages of the project, they do not support a formal analysis of change over time. Comparative analysis supported the identification of recurring themes while preserving setting-specific context (Yin, 2009), strengthening analytic robustness and theoretical transferability. In total, the study comprised 40 one-to-one interviews, focus groups with 15 staff, and repeated focus groups with 264 autistic pupils, alongside open-text responses from 164 pupils. Comparisons were made between pupils attending SRCs, mainstream pupils in SRC-hosting schools (MSRC), and pupils in schools without SRCs (NSRC).

**Table 1.** Data Collection Schedule.

Time point	Academic year	Data collection activities
1	Beginning of Year 1	• Focus groups with autistic pupils
2	End of Year 1	• Focus groups with autistic pupils • One-to-one interviews with autistic pupils in Year 10 • One-to-one interviews with parents and staff
3	Beginning of Year 2	• Focus groups with newly enrolled autistic pupils in Year 7
4	End of Year 2	• Focus groups (and qualitative survey responses) with autistic pupils
5	End of Year 3	• Focus groups (and qualitative survey responses) with autistic pupils • One-to-one interviews with parents and their child • Focus groups with Heads of Centres

### Participants and Procedures

#### Autistic Pupils

##### Pupil Interviews

Semi-structured interviews were conducted at Time 2 with 12 autistic pupils in Year 10 (aged 14–15 years) (7 male; 4 female; 1 other) (9 White; 1 Asian; 2 mixed-race) across three school settings (7 SRC; 1 MSRC; 4 NSRC). All participants had an Education Health and Care Plan (EHCP), a statutory document stating a child or young person’s educational, health, and social care needs and the support required to meet them. Interview durations ranged from 10 to 31 min (*M* = 17:58) and explored support use, peer relationships, and identity.

##### Focus Groups with Autistic Pupils

In total, 264 participants in Years 7–10 (ages = 11–15) took part at one time point or more across three school years. Overall, 112 (42%) had an EHCP: all SRC pupils (79/79; 100%), 24/120 (20%) in MSRC, and 9/65 (13.8%) in NSRC. Focus groups were conducted at Time 1 – Time 5 (T1–T5) in quiet, familiar rooms (20–30 min; 2–8 pupils in each). Pupils were asked about their school year, the school environment, coping/problem-solving (T1 and T2), peer relationships and the sensory environment (T3 and T4), and accessibility together with academic and emotional support (T5). Focus groups were not audio-recorded; instead, one researcher facilitated the session while another took detailed notes during and immediately following sessions to capture participants’ phrasing, key examples, and points of consensus or disagreement.

##### Open-Text Responses

A subset of the focus group pupils from three mainstream secondary schools (*N* = 164; 117 male; 40 female; 5 non-binary; 2 gender undisclosed) also provided open-text responses in an online survey at T4 or T5. The sample included 56 pupils based in an SRC, 80 in MSRC, and 28 in NSRC. Participants were drawn from Years 7 to 10 (aged = 11–15) (21 in Year 7; 57 in Year 8; 58 in Year 9; 28 in Year 10). Open-text responses were collected via pupil surveys at T4 and T5 using school devices. Pupils responded individually to two open-ended questions: (1) “What do you like most about school?” and (2) “What changes would make school a better place for you?” *Qualtrics* Surveys were administered in a quiet, familiar setting on school devices with researcher support with reading or comprehension as needed – typically in groups of 1 to 10, without discussion.

#### Parents of Autistic Pupils

Ten parents (nine mothers, one father) of autistic pupils were recruited through participating schools via indirect invitation. Letters containing study information and a consent link were sent to parents of autistic pupils with an EHCP; parents voluntarily provided informed consent directly to the research team via Qualtrics, thereby maintaining their anonymity. All parents were based in South-East England and had children enrolled in mainstream secondary schools. Five parents had children attending SRCs, two had children in MSRC, and three had children in NSRC. At Time 5, follow-up interviews were conducted with five of the 10 original parents. Their child was invited to take part in an individual interview. Three children consented to participate – one with their parent and two individually (see Table 2 for detailed demographics). Semi-structured interviews were conducted remotely via Microsoft Teams. Interviews explored perspectives on their child’s educational experience, including transitions, wellbeing, aspirations, relationships, and family impact. T2 interviews ranged from 27 to 57 min (*M* = 36:12), and T5 interviews ranged from 31 to 52 min (*M* = 40:25).

**Table 2.** Participant Demographics by Setting Type – Interviews With Parents.

Child setting	*n* (parents)	*n* (children)	Parent gender	Parent age range	Parent ethnicity	Parent employment status	Child gender	Child year group (range)
*SRC*	5	6	5F 1M	30–49	4 White, 1 Mixed	3 Self-employed, 1 Employed PT, 1 Unemployed	3F 2M	7–10
*MSRC*	2	2	2F	40–49	2 White	2 Self-employed	1F 1M	7–8
*NSRC*	3	3	3F	30–49	3 White	2 Employed FT, 1 Employed PT	1F 2M	7
*Total*	10	11	10F	30–49	9 White, 1 Mixed	5 Self-employed, 3 Employed, 1 Unemployed	6F 5M	7–10

PT part time; FT = full time; F = female; M = male.

Data are presented in aggregate by child setting to protect participant anonymity. Phase 2 child interviews: SRC – 1 pupil interviewed; MSRC – 1 pupil interviewed; NSRC – 1 pupil interviewed.

#### Staff

Fifteen members of school staff took part in individual semi-structured interviews at Time 2. Initial contact was made with Heads of Centres, inviting them to be interviewed and asking them to forward information to one further member of SRC support staff (e.g., specialist teacher/teaching assistants [TA]/learning support assistant [LSA]) and the school’s Special Educational Needs Coordinator (SENCO). In NSRC schools, invitations were sent to the SENCO, who was asked to forward the invitation to one additional member of SEN staff (Table 3). Staff interviews (ranging from 20 to 58 min; *M* = 38:37) were conducted in-person in school at T2, and tailored to role (operational structures, staffing, support strategies, transition planning, and collaborations). A remote focus group with all five Heads of Centre (86 min) at T5 explored operational practices, staff training, and the role of the SRC within their school.

**Table 3.** Participant Demographics by Setting Type – Interviews With School Staff.

School setting	*n* (staff)	Gender	Ethnic background	Job role category	Years teaching (range)	Years at current school (range)	Highest educational level
*SRC*	8	8F	7 White, 1 Mixed	Leadership: 4 Teaching: 2 Support: 2	6–28 (1 not recorded)	1.5–16	Degree level: 4 PGC/PGCE: 3 Master’s: 1
*MSRC*	4	4F	4 White	Leadership: 4	19–27	7–16	Degree level: 1 Master’s: 3
*NSRC*	3	3F	2 White, 1 Asian	Leadership: 2 Support: 1	8–27	2–4	Degree level: 1 Master’s: 2
*Total*	15	15F	13 White, 1 Mixed, 1 Asian	Leadership: 10 Teaching: 2 Support: 3	6–28 (1 not recorded)	1.5–16	Degree level: 6 PGC/PGCE: 3 Master’s: 6

F = female; M = male; PGC/PGCE = Postgraduate Certificate/Postgraduate Certificate in Education. Data are presented in aggregate by school setting to protect participant anonymity. Job role categories: Leadership = Head of SRC, SENCO, Deputy SENCO, and Senior Assistant Head Teacher; Teaching = Autism Teacher and Specialist Teacher; Support = Learning Support Assistant and Teaching Assistant.

### Ethics and Consent

Ethical approval was granted by the University of Surrey Ethics Committee. Accessible information sheets were provided. Written or verbal consent was obtained from adults; parental consent and on-the-day pupil assent were secured for young people. Participants could decline any question. Responses were anonymous, and pupils could withdraw at any time. Interviews were audio-recorded with permission and transcribed verbatim; focus groups were documented via structured researcher notes. All data were pseudonymised prior to analysis.

### Analysis

We employed reflexive thematic analysis for interview data (Braun & Clarke, 2006) to preserve analytic depth while enabling triangulation across sources. Each dataset (parents, staff, pupils) was analysed inductively in NVivo 14, with iterative coding and theme development based on analytical coherence and explanatory value rather than frequency. To enhance credibility, a second researcher independently reviewed a subset of coded transcripts, and discrepancies were resolved through discussion (Barker & Pistrang, 2005). We then conducted a cross-case comparative synthesis using an Excel matrix to map themes across stakeholder groups and setting type, interpreting patterns through Bronfenbrenner’s ecological systems theory, to identify convergent and divergent mechanisms shaping pupil experience (Glaser & Strauss, 1967; Stake, 2006). In parallel, conventional content analysis (Hsieh & Shannon, 2005) was applied to pupil open-text responses and focus-group notes. Codes were derived inductively, grouped into thematic categories aligned with question prompts, and summarised with prevalence counts. A second researcher reviewed a subset for reliability; discrepancies were reconciled. Findings were triangulated with interview-derived themes to strengthen interpretive validity (Denzin, 1978; Patton, 1999).

Reflexivity was considered throughout the analytic process. The longitudinal design involved repeated site visits over 3 years, resulting in sustained immersion in the participating school environments and datasets. Analysis was conducted collaboratively, with ongoing discussions between authors used not only to refine theme boundaries but also to interrogate how our respective perspectives shaped interpretation. For example, lived experience of neurodivergence and parenting informed sensitivity to issues of belonging, support, and relational fit, which were actively reflected on during coding and theme development to ensure that interpretations remained grounded in participants’ accounts rather than presupposed priorities. Input from National Autistic Society (NAS) colleagues further supported reflexive engagement by situating interpretations within broader policy and practice contexts, while also prompting critical reflection on how findings might be framed for applied relevance. While there was strong convergence in interpretation due to consistent patterns across schools and datasets, these reflexive discussions were central to ensuring analytic rigour and transparency.

### Positionality Statement

This study was developed within a commitment to participatory and neurodiversity-affirming autism research. The first author is autistic and has ADHD, contributing an insider perspective on neurodivergent schooling, wellbeing, and the importance of relational and environmental fit. The second author is a parent of two autistic young people, bringing long-term lived experience of navigating educational provision, support systems, and school inclusion. These positions shaped our interest in evaluating SRCs within mainstream schools and in foregrounding autistic pupils’, parents’, and staff understandings of what enables belonging, learning, and flourishing across different provision models. We recognise that our identities and experiences may influence the questions we ask and the analytic emphases we bring to the work. The findings should therefore be understood as situated within U.K. educational settings and within ongoing efforts to improve inclusive practice.

### Participatory Methods

To strengthen accountability and centre autistic perspectives, we worked iteratively with NAS, a leading U.K.-based autism charity, throughout study design and interpretation of findings. During the design phase, NAS researchers provided feedback on the selection of quantitative measures and the development of qualitative interview schedules. Interview questions for autistic pupils were also piloted with two autistic young people to ensure clarity and suitability.

Participatory considerations also informed data collection. Prior to each annual school visit, researchers held planning calls with SENCOs to review the research protocol, minimise disruption to pupils’ routines, and identify any environmental or staffing adaptations needed to support participation. We also treated participants’ accounts over the 3-year period as a source of expertise, using their feedback to refine procedures and enhance accessibility.

Interim updates were shared with NAS partners during the project to discuss emerging observations and practical considerations from pupil and educator perspectives. While analytical decisions and interpretation of the final dataset remained the responsibility of the academic research team, consultation with NAS offered valued feedback to contextualise emerging results, reflect on the limitations within our data, and strengthen interpretation.

## Results

### Overview of Themes

**Figure 2.**
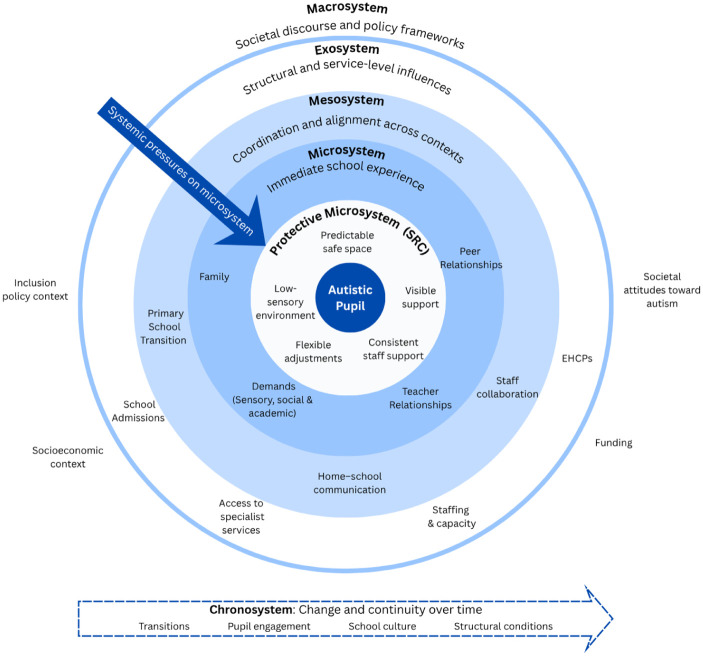
Ecological model of autistic pupils’ school experiences: mapping key factors across systems.

Analysis across pupils, parents, and staff identified four overarching themes: (1) *School environment and adaptations*, shaping pupils’ ability to engage and feel safe; (2) *Identity, belonging and emotional wellbeing*, highlighting anxiety, masking, and bullying; (3) *Supportive relationships and consistency*, capturing the protective nature of trusted, knowledgeable adults, and clear home-school communication; and (4) *Systemic pressures on the microsystem*, which shifted burdens onto schools and families and constrained inclusion. Figure 2 presents an ecological mapping of key factors identified in the analysis, illustrating how influences across system levels relate to autistic pupils’ school experiences.

### Theme 1: School Environment and Adaptations (Microsystem)

The school environment emerged as a central influence on autistic pupils’ ability to engage, regulate, and feel safe. Physical and structural features within schools were not merely contextual but woven together with pupils’ overall wellbeing and access to learning. This theme explores how environmental design, access to resources, and the visibility and flexibility of support systems either enabled or undermined pupil wellbeing.

#### Sensory and Emotional Regulation

The mainstream environment was frequently described as overwhelming. Pupils and parents cited crowded corridors, noisy canteens, and visually busy classrooms as barriers to learning and comfort, with one parent concerned about: “the trauma experienced by sitting in a classroom of that size” (Parent 5 – MSRC). Sounds from peers talking or teachers raising their voices were common stressors, with some pupils reporting shutdowns during lessons (i.e., being temporarily physically/emotionally frozen due to overwhelm). SRCs offered structured mechanisms for regulation that buffered cumulative stress: “they very much appreciate the safe space aspect to retreat to when life becomes too much . . . having somewhere that’s constantly available to them if needed is vital” (Head of Centre 2). Centres had dedicated sensory rooms, quiet areas, and sometimes animals and could be accessed at predictable points in the day to settle and decompress, with reliance often decreasing over time: “I used to come every day at break and lunch . . . Now I don’t come as much because I spend time with my friends in mainstream” (Pupil 5 – SRC Female). Across accounts, these spaces functioned not simply as quieter environments but as mechanisms for interrupting escalation, enabling pupils to regulate before distress became unmanageable, thereby supporting continued participation in the school day. Across timepoints, some pupils described reduced reliance on the SRC as confidence increased, suggesting a gradual shift from dependence towards more independent participation in mainstream contexts. However, this pattern was not universal, with others continuing to rely on the centre as a consistent source of regulation and support. For several pupils, tangible calming resources mattered: “he had a little tent he could go in and calm down . . . it’s a place that he feels he knows, and it’s safe for him” (Parent 3 – SRC).

#### Accessible Infrastructure and Physical Spaces

The physical infrastructure offered practical advantages. Separate toilets and private changing areas for physical education lessons alleviated a major source of distress: “if it wasn’t for a toilet, I bet you at least half of the students wouldn’t be using the toilet at all during the day” (Head of Centre 1). Sitting tests in quiet, familiar rooms improved focus: “I just have tests here as it is a quieter environment compared to the clicking of pens, the coughing, the sneezing” (Year 10 Male – SRC). A quieter lunch space helped pupils who otherwise found lunchtimes unmanageable: “she wasn’t eating in the day . . . the fact that she can go there makes a difference” (Parent 5 – MSRC). Such adjustments were reported more consistently within SRCs but were also offered to some MSRC pupils. These adjustments reduced routine points of friction that otherwise accumulated across the day and eroded pupils’ capacity to participate in school.

#### Flexible Support and Adaptive Learning

Inflexible policies were often a source of stress. Parents described uniform rules that didn’t account for sensory sensitivities and could trigger sanctions: “He doesn’t like his top button done up . . . but a teacher kind of got hold of this and put him in detention” (Parent 10 – NSRC). They also reported rigid behaviour policies: “she follows the rules to the nth degree . . . in case she forgets a pen you know because that’s an automatic detention” (Parent 5 – MSRC). In contrast, flexible timetables, reduced homework, and subject adjustments were protective: “he’s now only required to do his core subjects homework . . . because that was making things quite stressful” (Parent 9 – NSRC). Across accounts, the issue was less the presence of rules per se than the failure of blanket rules to distinguish between deliberate non-compliance and needs arising from sensory, cognitive, or emotional differences. In this sense, inflexibility did not simply withhold support; it actively produced avoidable distress.

#### Visibility, Approachability, and Accessibility of Support

Across settings, schools used adaptations such as exit cards, toilet passes, and queue-jump cards, but pupils’ awareness of and confidence in using these varied. In NSRC schools, parents and staff were particularly likely to describe inconsistency in how agreed adjustments were implemented, with parents reporting that pupils were sometimes questioned when attempting to use them “she has used it [an exit card] but has been questioned on that by some teachers” (Parent 8 – NSRC). Staff similarly acknowledged that agreed accommodations and arrangements were not always applied consistently, with this practice “penalising those children for having a difficulty or requiring extra time” (NSRC Staff – 14). By contrast, pupils tended to describe the practical consequences of this inconsistency, including hesitating to use adjustments unless absolutely necessary.

In SRC contexts, support-seeking appeared more normalised: “I do come here when I’m overwhelmed . . . need to let some things off my chest and that and I find it helps” (Pupil 7 – SRC Male). Staff also emphasised the role of whole-school practices in reducing stigma; in one centre, for example, the term “intervention” was deliberately applied to all pupils leaving class for additional support “so it’s not different” (Head of SRC – P4), meaning that participation in support activities did not mark pupils as different. However, some NSRC pupils described perceived inequities in access to support, with one pupil expressing frustration “I feel a bit jealous honestly . . . it feels like I’ve been forced to not get support while still struggling” (NSRC Male – Year 9).

Across cases, the key difference was less whether adjustments existed than whether they were predictable, visible, and socially safe to use.

### Theme 2: Identity, Belonging, and Emotional Wellbeing

Autistic pupils’ identity, belonging, and emotional wellbeing were tightly linked and often fragile in mainstream schools. Overwhelming environments and social demands affected mental health and shaped how pupils saw themselves, with many internalising difficulties as personal failings. High anxiety, masking, and burnout sometimes led to school non-attendance, social withdrawal, and dysregulation, particularly at home. This theme examines how relationships, stressors, masking, and evolving friendship dynamics interact to shape identity, wellbeing, and belonging.

#### Peer Attitudes, Stigma, and Social Inclusion

Bullying and negative peer attitudes were common concerns. Pupils described how autism is used as a slur: “it’s not generally treated as a positive thing like it’s sometimes used as an insult” (Pupil 6 – SRC Male). Accounts highlighted how stigma operated not only through overt bullying but also through the broader social positioning of autism as something treated as a joke, undesirable, or risky to disclose. Staff recognised this issue: “I’ve heard kids say ‘oh you’re autistic’ like it’s an insult and that needs to be stamped on” (NSRC Staff 14). Peer attitudes shaped not only how pupils were treated but also how cautiously they managed their visibility within school. Many pupils avoided reporting peer behaviour, adopting solitary coping and social withdrawal (e.g., spending breaks in the SRC or SEND area). Although incidents of bullying tended to reduce with age, this was not universal – with many pupils expressing a sense of resignation: “in the secondary school environment it [autism] really just feels like kind of a weak spot” (NSRC Male – Year 9).

#### Friendship Dynamics, Social Motivation, and Navigating School Social Life

Friendships were important and pupils valued kindness, loyalty, and humour, but dynamics could be complex. SRC pupils often formed close friendships within the centre’s socially safer environment: “Her friends are people in the Centre . . . that’s who she’s spending time with out of lessons” (Parent 1 – SRC). A pupil reflected: “it’s not full of loads of people, it’s just some and we’re all kind of similar” (Pupil 4 – SRC Female).

NSRC pupils more often sustained long-term mainstream friendships and socialised more outside school. However, parents of non-SRC pupils highlighted the risk of over-reliance on a single “safe” friend; one parent noted that their daughter “*won’t walk to school if that friend is off sick*” (Parent 5 – MSRC). In these cases, schools sometimes intervened by adjusting seating plans or group work to ensure trusted peers were nearby. SRC contexts appeared to offer an enclosed but often more accepting peer network, whereas pupils in non-SRC settings more often relied on long-standing mainstream friendships that could be protective but also fragile.

#### Masking and Autism Disclosure

Across accounts, masking functioned as a social survival strategy, as one pupil said, “if I didn’t mask, I think I’ll probably more likely get made fun of” (MSRC Girl – Year 10). However, while removing immediate visibility in school, it simultaneously obscured needs from adults: “I’m quite good at hiding things . . . the teachers just have no idea. Some are just completely oblivious to what’s going on” (Pupil 11 – NSRC). The emotional cost of this practice was high and was frequently displaced, manifesting as exhaustion or dysregulation at home: “When she gets home . . . she’ll be really grumpy and shout and things, which is obviously really difficult” (Parent 8 – NSRC). Parents most often described the after-school consequences of masking, while pupils were more likely to describe the effort involved in performing coping at school. Among staff, SRCs were recognised as: “a place to come to unmask” where pupils could “be their authentic selves” (Head of Centre). Outside centres, opportunities to drop the mask were fewer, leading to cumulative strain.

#### Mental Health Challenges: Anxiety, Exhaustion, and Burnout

Across settings, anxiety, exhaustion, and burnout were pervasive, particularly among girls. Importantly, these difficulties were rarely described as discrete mental health problems separable from the school context; rather, they were presented as cumulative outcomes of sustained social, sensory, and performance demands within the school environment. Parents described anxiety, low mood, fear of failure, and time spent recovering at home: “a lot of time spent in bed and with like the curtains drawn, which I think is partly a way of managing everything that’s to be done in the day” (Parent 2 – SRC); “she does vomit sometimes with anxiety . . . fear of failure, which is a big thing for her” (Parent 1 – SRC). The problem was most prevalent in schools without an SRC, where there was little respite from the aforementioned demands. One parent reflected, “she’s definitely not the child that she was before” (Parent 8 – NSRC) reflecting how sustained school stress was experienced not as episodic difficulty but as something that gradually reshaped pupils’ wellbeing, character, and development. Across accounts, these experiences were often described as developing over time rather than as isolated incidents. While some pupils showed improved coping or social integration, others experienced increasing anxiety and exhaustion, suggesting that change was uneven and shaped by the consistency of support and environmental fit.

### Theme 3: Supportive Relationships and Consistency (Microsystem and Mesosystem)

Relational dynamics mediated how pupils experienced school demands, shaping safety, belonging and emotional regulation across settings. Trusted adults, consistent staffing, and clear communication were protective, helping pupils feel understood and manage school’s demands. While such relationships existed in all settings, they were more reliably described within SRC contexts, where communication was more proactive, and pupils felt more comfortable seeking staff support.

#### Trusted Adults and Consistent Staff Presence

Parents and pupils highlighted how even one reliable relationship with a staff member could transform daily experience. Pupils and parents valued staff who knew their profile and intervened early: “someone in their corner” who “gets it.” A parent described how the school counsellor was “critical as part of how it’s going in school . . . she’s usually the one who really steps in” (Parent 5 – SRC). Another highlighted anticipatory support: “Her TA’s very, very patient . . . she can see it coming and she will just divert it off earlier . . . so she’s processed quite a lot by the time she arrives [home]” (Parent 1 – SRC). Pupil preferences were also considered when allocating SRC staff support, helping maintain continuity and reduce barriers to accessing education. However, it was not only the presence of support that mattered but also *consistency* in who provided it. Staff turnover across all settings could undermine the sense of security that familiarity brings.

#### Staff Understanding and Awareness

Staff awareness and application of autism knowledge varied. In NSRC schools, staff reported inconsistent use of reasonable adjustments, for example, colleagues’ mismanagement of extra time in exams: “children are expected to have their extra time ‘and it might run into break but that’s okay’ . . . er no it’s not!” (NSRC Staff 14). Analysis of pupil open-text responses showed a higher proportion of NSRC than SRC pupils identifying the need for a “safe and respectful community,” often related to teachers being understanding and listening. One pupil described teachers not understanding their way of processing: “I get like ‘zone out stage’ and they just think that I’m not doing the work and like, no I know I’m taking everything in – I’m just in one of those hyper-fixation modes where I just don’t respond” (Pupil 11 – NSRC). In SRC schools, nuanced understanding and proactive identification were more common: “[Head of Centre] recognised that she should be . . . taken to the doctor and see if she doesn’t have ADHD . . . it was actually quite easy to see” (Parent 4 – SRC). In contrast, in NSRC settings, adjustments depended heavily on individual staff members rather than whole-school practice: “That would be the ideal . . . but I don’t think that necessarily always happens” (NSRC SENCO – P13).

#### Communication Channels Between School and Home

Across all settings, the quality of home-school communication hinged on having clear, accessible points of contact. For SRC pupils, this role was typically the Head of Centre, who could cascade key information to the wider staff team and pre-empt challenges before they arose. Among NSRC schools, this role varied – sometimes a tutor, SENCO, or counsellor – however, communication was generally more reactive and dependent on proactive parents initiating contact. Parents in SRC settings described a more responsive, proactive approach. One SRC parent noted: “I’ve got a really good relationship with the staff there . . . You know, it’s a text message, e-mail, whatever’s needed. Give her a ring ‘I really need to talk to you’. She’ll phone me back” (Parent 2 – SRC). In NSRC settings, contact felt more reactive: “quite a battle actually, to talk to school about her learning” (Parent 8 – NSRC). Some parents contacted individual teachers to negotiate short-term flexibility: “I kind of emailed the maths teacher saying he’s really anxious at the moment . . . Can you be lenient with him?” (Parent 6 – MSRC). Staff noted that pupils whose parents lacked the capacity or confidence to advocate were particularly disadvantaged: “we do have some parents of kids with ASD that really struggle” (NSRC Staff 14) and “Parental anxiety’s very high . . . they can then in turn put a lot of pressure on teachers, which increases the teacher anxiety” (Head of SRC – P2).

#### Collaboration Among Teachers, TAs, and Specialists

Analysis of staff interviews reflected how collaboration was strongest where the SRC ethos permeated the whole school. SRC teams maintained open information channels and joint problem-solving forums, paired with ongoing staff training and continuing professional development (CPD) that embedded neuro-affirming strategies to support autistic pupils and reframe behaviour: “we talk to the teachers about behaviours as a form of communication” encouraging staff to “unpick why they’re seeing these behaviours” (Head of SRC – P3). Staff accounts, particularly from Heads of Centre, pointed to gradual shifts over time in whole-school understanding of autism, with increased staff confidence, more consistent use of inclusive practices, and greater engagement from mainstream teachers, who increasingly sought advice from centres: “We’ve actually seen more mainstream staff coming into the [SRC] now to talk to us about individual students . . . finding out what our advice would be when strategies aren’t working” (Head of SRC – P2). However, such changes were described as incremental and uneven across settings. Outside of SRC contexts, collaboration depended on individual goodwill and leadership buy-in: “So many teachers are amazing . . . but it doesn’t help when it’s not being reiterated from the top” [NSRC Staff 14]. The analytic distinction, then, was not whether collaboration occurred at all, but whether it was formalised, routinised, and leadership-supported rather than dependent on individual enthusiasm.

### Theme 4: Systemic Pressures on the Microsystem

System-level deficits – staff shortages, scarce specialist services, and delayed diagnoses – place sustained pressure on schools and families with consequences felt most acutely at the microsystem level. Although rooted in wider exosystem/macrosystem factors (e.g., local authority processes, national policy, societal attitudes), the impact is felt sharply at the individual level, with parents and teachers absorbing the emotional and behavioural burden beyond their capacity.

#### Staffing Shortages and Training Needs

Across stakeholder accounts, staffing instability mattered not only as a workforce issue in itself but also because it disrupted the continuity, familiarity, and autism-specific understanding that pupils relied upon. Recruitment and retention problems were widespread, especially for TAs: “trying to get decent TA’s back in is really tricky . . . you can get paid more if you go work in a supermarket” (Head of SRC – P4). Shortages increased reliance on substitute teachers, disrupting consistency and undermining specialist knowledge and presenting challenges in maintaining continuity of CPD: “you can’t always be sure that those supply teachers have an up-to-date and practical knowledge of how to support autistic young people” (MSRC SENCO – P9). Pupils often found continuous supply teachers disruptive, opting to use the SRC during cover lessons to reduce the unpredictability and routine-disruption caused by staffing turnover: “when things are different, I didn’t like it” (Pupil 1 – SRC). In contrast to some of the relational and cultural shifts described elsewhere, these systemic challenges were consistently reported across timepoints, suggesting limited change in underlying structural conditions over the duration of the study.

#### Societal Attitudes Towards Autism

Wider social narratives about autism shaped everyday interactions in school, influencing how pupils, staff, and families approached identification and support. Staff noted how parents were sometimes reluctant to seek assessments, and some pupils were unaware of their own diagnosis. Pupils often chose not to disclose their autism due to autism being discussed negatively or as a joke. However, schools attempted to shift attitudes through training and assemblies. One head described a gradual culture change:

There’s an awful lot of practice that now happens in the whole school and . . . it’s just become a natural thing like breathing . . . little everyday things like having an understanding of why a child cannot stand in a queue . . . whereas that might have been a big issue previously now it’s just second nature that they understand the reasons why that child’s finding that queue difficult. (Head of SRC – P2)

#### Missed Early Diagnoses and Unmet Needs in Primary

Needs were sometimes misinterpreted in primary school, with behaviours viewed through a disciplinary lens rather than indicators of unmet need. Parental and staff accounts highlighted how secondary placement and support were often shaped by patterns of earlier non-recognition. One parent described how during primary school their child was: “very disruptive in the classroom” meaning “his potential wasn’t actually tapped into because they were just trying to deal with the behaviour” (Parent 6 – MSRC). Other pupils masked so effectively that needs went unnoticed, with one parent recalling how their child: “did a brilliant job of masking everything she found hard . . . didn’t stick her head above the parapet” (Parent 7 – MSRC). Delays in EHCPs had knock-on effects for transition and SRC admissions. One head of centre shared her frustration: “This coming year 7, starting in September, I have got zero [SRC] students coming . . . only one of them has an EHCP . . . primary schools just don’t think ahead. They’re very much ‘well they’ve coped so far’ – over to you!” (Head of SRC – P1). As a result, many pupils were transitioning into secondary school already carrying the strain of cumulative unmet needs, and without appropriate planning, so that pupils are given a suitable placement.

#### Limited Availability of Appropriate Mental Health Support

Access to autism-informed mental health services was constrained by waiting lists and eligibility criteria. Schools were left absorbing the shortfalls: “when social, emotional and mental health (SEMH) becomes the most presenting need . . . we feel really stuck because they desperately need specialist therapeutic support and we aren’t able to do that within the resources that we’ve got” (Head of SRC). This left schools in the position of managing need without the remit or specialist capacity to treat it. Even when services did exist, they often couldn’t meet pupils’ needs. One Head of Centre explained, “they will pretty much always say they can’t work with neurodivergent students because they [the mental health support staff] have to be at a senior level” (Head of SRC – P1). Parents similarly voiced frustration that pupils whose distress was internalised or masked were at particular risk of being judged insufficiently severe for support: “She’s a child with autism, who masks constantly and [the Child & Adolescent Mental Health Service] couldn’t read through that” (Parent 1 – SRC), resulting in missed needs and premature discharge from services. These gaps also left staff feeling helpless and placed additional strain on families, including adapting or reducing work hours to accommodate part-time school timetables or unpredictable attendance.

#### Lack of Appropriate Placements and Specialist Resources

Across cases, schools reported persistent gaps in access to specialist therapies – particularly Speech and Language Therapy (SLT) and Occupational Therapy (OT). “Access to specialist therapies is quite limited” (Head of Centre – P4) with centres often having to buy in private provision or resort to online resources: “it’s working really well, but we are having to pay for it privately” (Head of Centre). In parallel, the admissions process – managed by local authorities was a source of significant strain. Heads of Centre and SENCOs described an “extremely high volume” of requests to consider pupils for placements outside the usual annual admissions cycle, often for the admission of pupils whose needs could not realistically be met in mainstream. These placements required substantial additional resources that Centres did not have, and for some pupils, resulted in a lack of integration into mainstream school life: “that’s just not a good situation to be in because [they’re] . . . quite isolated from peers . . . They’re usually at some sort of crisis point, but we just end up with them here because there’s nowhere else for them to go” (Head of Centre – P2). Heads of Centres’ professional expertise was overridden by decisions made by local authorities, meaning that centres were required to meet the needs they were neither funded nor staffed to support. These accounts suggest that the value of SRC provision was contingent rather than universal: where placement fit and resourcing were poor, centres risked shifting from functioning as bridges into mainstream participation to acting as containment spaces for unmet needs at the exosystemic and macrosystemic levels.

Across the dataset, change over time was explored but was not a dominant pattern in participants’ accounts. Experiences were more often characterised by continuity than transformation. Where change was evident, it tended to be localised – such as gradual shifts in school ethos, evolving use of support spaces, or changes in individual engagement and wellbeing – rather than systemic change across settings. This suggests that while aspects of pupils’ experiences are dynamic, many of the structural and relational conditions shaping inclusion remain relatively stable over time.

## Discussion

This study examined what shapes autistic pupils’ experiences in secondary schools with SRCs and comparable mainstream schools without such provision. Extending prior qualitative and survey-based work (Frederickson et al., 2010; Hebron & Bond, 2017), our multi-school comparison foregrounds how environmental structures, relationships, and wider system conditions interact to enable or constrain inclusion. Viewing the findings through this ecological lens highlights how the effectiveness of support for autistic pupils cannot be understood solely at the level of classroom practice. Instead, pupils’ experiences reflected interactions across multiple layers of the school ecosystem. SRC environments often functioned as stabilising microsystems that legitimised needs and normalised help-seeking, providing predictability and relational safety. However, their effectiveness depended on mesosystem processes such as collaboration between staff and families and was sometimes constrained by exosystem pressures including staffing capacity, resource allocation, and wider service availability. In contrast, mainstream-only adaptations tended to be more ad hoc or contingent on individual staff, illustrating how cross-cutting systemic pressures limited what any school could sustain. This uneven pattern suggests that change over time – the chronosystem dimension – is not uniform across system levels or school contexts, with proximal supports (particularly within SRC provision) showing some adaptability while wider structural conditions remain comparatively stable across settings.

### SRCs as Protective Microsystems

Consistent with previous studies, SRCs were described as predictable, quieter spaces with clear routes to support, offering pupils a flexible “safe base” during the day (Bond & Hebron, 2016; Halsall et al., 2021; Landor & Perepa, 2017). At a practical level, concrete adjustments mattered: separate toilets and changing spaces, familiar rooms for examinations, and quieter options at lunch reduced everyday sensory and social burdens and enabled participation. Our comparative design highlights that these adjustments were more reliable and visible in SRCs than elsewhere. Beyond physical features, the symbolic presence of the centre helped pupils know where to go and when, particularly during transitions into secondary school. Many pupils described heavier early use of the centre followed by gradual, self-directed integration into mainstream spaces as confidence increased, echoing work showing SRCs can scaffold participation over time (Goodall, 2018b; Humphrey & Symes, 2011; Roberts & Simpson, 2016).

Socially, the SRC often provided a safer peer network, supporting belonging and friendships that might otherwise be difficult to establish in crowded mainstream settings (O’Hagan & Hebron, 2017; Warren et al., 2020). This was not universally positive: for some pupils, over-reliance risked limiting broader participation, underscoring the need for careful planning so the centre functions as a bridge rather than a boundary. Importantly, these patterns appeared to develop incrementally rather than rapidly, reinforcing the need to understand inclusion as a process unfolding over time rather than a fixed outcome.

### Trusted Relationships and Consistency

Relational dynamics were among the strongest protective factors. Pupils and parents consistently emphasised the impact of having at least one trusted adult who knew the pupil’s profile and could anticipate difficulties, consistent with evidence that relationships with knowledgeable adults support regulation and belonging (Frederickson et al., 2010; Hebron & Humphrey, 2014; Symes & Humphrey, 2011). In our data, SRCs were better able to provide continuity and preference-matching between pupils and staff and to normalise support-seeking. Communication with home also tended to be more proactive and pre-emptive in SRC contexts, aligning with prior reports that resource bases may cultivate a stronger ethos of mutual support than mainstream-only models (Bond & Hebron, 2016; Croydon et al., 2019; Hebron & Bond, 2017). Where mainstream-only schools succeeded, it was typically through individual staff commitment; where they struggled, parents described reactive or fragmented communication.

Policy flexibility was another crucial ingredient. Flexible timetables, adapted homework expectations, and reasonable adjustments that were understood and consistently honoured helped pupils participate; conversely, rigid uniform and behaviour rules created avoidable anxiety. These findings corroborate previous studies suggesting that SRCs can offer greater flexibility to meet individual needs and support belonging and academic participation (Frederickson et al., 2010; Goodall, 2019; Roberts & Simpson, 2016). Notably, many mainstream-only schools had similar tools on paper (e.g., exit cards), but pupils’ knowledge of and confidence in using them varied, and teachers sometimes questioned their use in the moment. This underscores the importance of whole-school consistency and everyday language and routines that normalise adjustments.

### Systemic Challenges

Despite best efforts, several system-level constraints curtailed impact. Notably, these constraints were described consistently across timepoints, indicating limited change in wider system conditions over the duration of the study. Admissions processes sometimes placed pupils in SRCs whose needs could not realistically be met in mainstream, stretching resources and, for a minority, resulting in minimal engagement with mainstream schooling. Unmet demand for specialist therapies led some centres to buy in private provision or go without. Workforce issues were pervasive: recruitment and retention problems, especially for TAs, increased reliance on supply cover and disrupted continuity, echoing wider workforce and commissioning issues reported nationally (National Audit Office [NAO], 2019) and internationally (Renty & Roeyers, 2006).

Mental health need was a recurring thread. Anxiety, burnout, and emotional-based school non-attendance were widely reported, particularly among girls, consistent with elevated internalising risks documented in the literature (Ashburner et al., 2010; Hebron & Humphrey, 2012; Ridgway et al., 2024; Moyse & Porter, 2015; Van Steensel et al., 2011; King et al., 2020). In our sample, SRC placements appeared to reduce attendance gaps relative to national patterns, suggesting a mitigative role for some pupils (Cook & Boddy, 2026). However, limited access to autism-informed mental health services left schools feeling “stuck” and families absorbing the fallout, echoing broader critiques of provision gaps and thresholds (Ashworth et al., 2025; Hansen et al., 2021). From an ecological perspective, these pressures illustrate microsystem-macrosystem tensions: pupils expended effort to appear to cope at school (masking) while dysregulation was displaced to the home mesosystem; negative peer/societal narratives shaped disclosure and help-seeking, while exosystem service constraints set limits on what schools could achieve alone. This pattern also highlights the chronosystem dimension, whereby pressures accumulate across time, with the effects of masking, unmet need, and limited support often becoming more pronounced rather than resolving.

### Implications for Practice and Policy

First, inclusion requires visible, reliable routes to support. The elements that distinguished SRCs from mainstream-only provision in practice were predictability, clear points of contact, normalised adjustments, and calm spaces to decompress. Schools without SRCs can still enact these principles by establishing visible safe spaces, ensuring understanding and honouring of adjustments across staff, and designating a consistent adult who coordinates support and home communication.

Second, flexibility should be built into rules and routines. Behaviour and uniform policies that allow for sensory, cognitive, and emotional differences can reduce avoidable distress. Similarly, adjusted homework expectations, pragmatic timetabling, and access arrangements that do not inadvertently penalise pupils are low-cost ways to protect engagement.

Third, investment in staff capacity and continuity is central. Ongoing autism-informed CPD, opportunities for joint problem-solving, and leadership endorsement of SEND priorities help translate knowledge into consistent practice. Given turnover realities, modular CPD with refresh cycles may be necessary to sustain skills.

Fourth, system coordination matters. Clearer admissions criteria and pathways, timely EHCP processing, and access to autism-competent mental health support would reduce crisis-driven placements and help schools use resources as intended. Inter-agency protocols that recognise masking and internalising presentations are likely to improve identification and support.

### Strengths, Limitations, and Future Directions

Strengths include the comparative, multi-school design; triangulating perspectives from pupils, parents, and staff offered a more holistic account than prior single-site or single-perspective studies, helping to illuminate convergences and divergences across layers of the school system. Limitations include regional concentration within one SRC network, which may restrict generalisability, and potential mismatch between adult accounts and pupils’ internal experiences (a known issue in camouflaging research; Halsall et al., 2021).

Future research should examine temporal dynamics more explicitly, including transitions at Year 6–7 and post-16, and the conditions under which change occurs over time across different system levels. Work is also needed on how SRCs function as inclusive services for the whole school (e.g., outreach, teacher forums, on-call coaching) versus as separate spaces, and on the design of school-level interventions that preserve the protective elements identified here while avoiding over-reliance. Finally, a stronger linkage between education and mental health services is essential, with evaluation of autism-informed models that can be delivered at scale without displacing need back onto schools and families.

## Conclusion

Inclusion is not determined by structure alone. Across settings, pupils’ participation and wellbeing were shaped by the interplay of environment, relationships, and system capacity. SRCs often provided a protective microsystem that made support visible, predictable, and acceptable; where their ethos permeated the wider school, pupils reported greater belonging and engagement. Yet the same themes recurred everywhere: small, concrete adaptations matter because they mitigate sensory and social burdens; trusted adults matter because they anticipate and coordinate help; and system conditions matter because they set the ceiling for what schools can sustain. Over time, effective inclusion, therefore, depends on aligning these layers so that everyday practice is both compassionate and structurally supported, with policy and services enabling schools to meet needs rather than shifting them elsewhere.

## Supplemental Material

sj-docx-1-aut-10.1177_13623613261457949 – Supplemental material for Specialist Resource Centres as Protective Microsystems: A Qualitative Comparative Case Study of Autistic Pupils’ Experiences in Mainstream Secondary Schools Supplemental material, sj-docx-1-aut-10.1177_13623613261457949 for Specialist Resource Centres as Protective Microsystems: A Qualitative Comparative Case Study of Autistic Pupils’ Experiences in Mainstream Secondary Schools by Alice Boddy and Anna Cook in Autism
