# Supplementary material for: Specialist Resource Centres as Protective Microsystems: A Qualitative Comparative Case Study of Autistic Pupils’ Experiences in Mainstream Secondary Schools
Source: Autism. 2026 Jul 2;30(8):2079–94. doi: 10.1177/13623613261457949 (PMC13392175; doi:10.1177/13623613261457949)
Supplement: sj-docx-1-aut-10.1177_13623613261457949 – Supplemental material for Specialist Resource Centres as Protective Microsystems: A Qualitative Comparative Case Study of Autistic Pupils’ Experiences in Mainstream Secondary Schools [file sj-docx-1-aut-10.1177_13623613261457949.docx]

**SUPPLEMENTARY MATERIALS**

**Interview and Focus Group Guides**

*[All interviews and focus groups began with an introduction to the research team, a clear explanation of the study purpose and topics, and information about confidentiality, anonymisation, and data use. Participants were reminded that participation was voluntary, that they could decline to answer any question or withdraw at any time, and that sessions would be audio-recorded with consent, where relevant. Opportunities for questions and clarification were provided throughout.]*

**Interviews with Y10 Autistic Students – Time 2**

**1. Your experience of learning in school**

1. How long have you been at this school?
2. Thinking about your school in general, if you had to sell it to someone who was thinking about coming here, what positive things would you tell them?
3. In contrast, what things would you wish to warn them about?
4. Can you tell me a bit about the lessons you like most and/or lessons you think you are good at?
5. *(If pupil mentions a particular subject they like)* So what is it about [*subject]* that you like and why do you think you are good at it?
6. Are there any lessons you don’t like and/or think you are not very good at, and if so, can you tell me why you don’t like them?
7. Do you have any lessons away from the rest of the class? If so, how do you feel about this?
8. Has the experience of learning changed as you moved up the school – particularly GCSE choices and experience of Y10.

**2. Your use of school support**

1. [SRC], can you tell me a bit about how you use the SRC?
   1. lesson times, break times, changing etc
2. [Others] how much do you make use of the specialist centre/SEN room? If you use it, in what ways do you use it?
3. How does the SRC/specialist centre/SEN room support you
   1. Facilities, resources, staff, environment, social space etc
4. What do you think of how your teachers treat you? (e.g. fair, understanding, respectful, reliable)
5. [SRC] do teachers treat you differently inside and outside of the SRC? If so, how?
6. Does a teaching assistant help you in any of your lessons, and if so, in what way do they help with your learning?
7. Do your classmates support you (a. with your work; b. with other parts of your day)
8. How do you feel about coming into school each day?

**3. About your friends**

- 1. Are you satisfied with the friendships you have at school?
  2. Please can you tell me how you feel you get on with people at school?
- Do you have any friends who you meet outside of school, and if so what kind of activities do you do together?
  1. If no:
- Do you have any friends outside of school? If so,
- What do you do with them?
  1. Are there any friends that you had in the past who you don’t see any more? If so, what happened?

**4. Your thoughts about whether you have been treated unfairly by fellow school pupils**

1. Have any pupils at school ever done or said something you didn’t like? If so, please can you tell me a bit about what happened?
2. *[If they answered yes…]* Why do you think those pupils treated you that way?
3. How did it make you feel?
4. Who helped to sort out the problem, and did that problem get sorted out?

**5. About autism**

1. (For SRC students only) Do you tell friends in the mainstream school that you have a place in the SRC? If so, are you proud to have a place? If not, why do you choose not to tell them?
2. Who in the school knows about your autism?
3. Do you tell friends that you are autistic? If so, how do they respond? Do you find this helpful? If not, why do choose not to tell them?
4. Do you think all of your teachers know you are autistic? If so, how does this help you? If not, do you think your school experience would be different if they did know/understand? How?
5. Some people are very happy with their autism diagnosis and others are less so. How do you feel about it?
6. Sometimes pupils may be having a bad day – e.g. you may get a bad test result, or someone has annoyed you. Can you tell me what kinds of things you would do to cope when you are feeling sad or frustrated or anxious?
   1. E.g., talk to someone, spend time with people who cheer me up, seek help from a teacher/TA, try to solve the problem on my own).

# **Student Focus Groups – Time 1, 2 & 3**

# **[**

# a. Most important things to you about school…

1. Please can you tell me a bit about your school year – what is the best and the worst thing about being at this school over the course of the year?

2. Are there any things in the survey that made you feel strongly?

(prompts: support from teachers; support from your peers; your sense of belonging to the school; your feelings/emotions; friendships; bullying)

If so, what were they and why?

# b. School Environment

3. We are interested in your views about the physical environment of your school – that is things like the building, its sights and sounds and smells etc.

I’d like to ask about 5 characteristics and for each one please can you think about it in relation to your school and what effect it might have on you [Only provide examples/ prompt if unsure]

a. visual characteristics – e.g., lighting, sunlight through blinds, reflection off surfaces, colours, signage, seating and wall decorations

b. Sounds – e.g., acoustics, noise levels, humming sounds of equipment, chairs scraping across floors

c. Touch – e.g., proximity to others during transitions/queuing, access to fidgets, seating plans, uniforms

d. Movement/body awareness – e.g., design of tables/chairs, stairs, surfaces, queuing

e. Smell – e.g. smell reduction, ventilation

**SRCs Only**

4. Please can you talk about the sensory differences between SRC and the rest of the school and how these affect you during your school day.

# c. Coping/problem solving

5. Sometimes we don’t always do so well in a test/piece of work.

If you receive a result that you weren’t happy with, can you tell me what you would do?

(prompts: find out what I did wrong; additional work/revision; ask for help with understanding material; ask a peer)

6. Sometimes we don’t feel 100% at school – we have days when we feel sad, frustrated or anxious.

On a bad day like this, can you talk about what you might do?

(prompts: talk to someone, spend time with people who will cheer me up, seek help from a teacher/TA; try to solve the problem on my own)

7. Do your actions make you feel better or worse? Why?

8. What are you most proud of this year – your biggest achievement?

# **Student Focus Groups – Time 4**

# a. Most important things to you about school…

1. Please can you tell me a bit about your school year – what is the best and the worst thing about being at this school over the course of the year?

2. What do you think about the SRC?

- Are there aspects of the physical space that you use or like the most?
- What kind of support do you receive from staff and how does this help you? For what reasons do you visit/use the SRC? Has this changed since last year?

# b. Peer relationships

1. I would like to ask you a few questions about your friendships this year at school
   1. Please can you tell me how you feel you get on with people at school?
   2. Is there anyone in particular that you like?

If yes [*prompts*]:

- What is it about them that you like?
- What is it that you think they like about you?
- What do you do with them?
- How did you come to be friends in the first place?

If no:

- Do you have any friends outside of school, and if so…. (as above)

1. Next, if you feel comfortable talking about it, please can you tell me if you have been treated unfairly by fellow pupils this year (e.g. saying hurtful things, leaving you out or hurting you)
2. If so, please can you tell me a bit about what happened?
3. *[If they answered yes…Prompt if necessary]*

- Who helped to sort out the problem, and did that problem get sorted out?
- Do these kinds of incidents happen often, and if so, how often (e.g. every day/every week/only once or twice)?
- Was there anything that caused the pupils to treat you that way?

# c. Sensory environment

1. Are there things about your school environment that make you feel stressed or anxious? If so, please can you describe them? [*Prompts:*]

- How does it make you feel?
- How does it affect your learning?
- Can you tell me how you find ways to cope with the feelings you have?

6. Are there any spaces/areas in the school environment that help you to relax or focus?

FINAL QUESTION:

7. What are you most proud of this year at school?

(This could be related to your school work, other things you do in your own time, or just something that has made you feel happy or content.)

# **Student Focus Groups – Time 5**

# b. School environment and accessibility

1. **Physical Environment**

- How do you feel about the classrooms and other spaces in your school? Are there any areas you find particularly comfortable or uncomfortable? Why?
- Are there any changes you would like to see in the school environment to make it more comfortable for you?

**2. (For SRC students only)**

- On an average day, how much time do you spend in the SRC (as a %)?
- For what reasons do you visit/use the SRC? Has this changed since last year?

2. **Sensory Considerations**

- How do noise levels, lighting, and other sensory factors in the school affect you?
- What does the school do well to support your sensory needs? Are there any improvements you would suggest?

3. **Accessibility of Resources**

- Do you find it easy to Access the resources and support you need at school (e.g., books, technology, support staff)?
- Are there any resources or supports you feel are missing or could be improved?

# c. Academic Experience and Support

1. **Classroom Learning**

- How do you feel about the way lessons are taught in your school? Are there teaching methods that work well for you? Are there any that don’t?
- Can you describe a time when you felt particularly Successful in your learning? What helped you do well here?
- What are your thoughts on how teachers manage behaviour in classes? Does this impact your learning?

2. **Individual Support**

- How do you feel about the support you receive from your teachers and support staff with your schoolwork?
- Are there any additional supports or changes you think would help you do better in your studies?

3. **Homework and Assignments**

- How do you manage your homework and assignments? Do you feel you receive enough support with these tasks?
- What changes, if any, would make completing homework and assignments easier for you?

# d. Wellbeing and emotional support

1. **Emotional Support**

- How do you feel about the emotional support you receive at school? Are there specific staff members or services that you find particularly helpful?
- Are you aware of any mental health resources available at your school
- Is there anything else you would like the school to do to help support students emotionally?

2. **Stress and Coping**

- What are some of the challenges or stressful situations you face at school? How do you usually cope with these challenges?
- Are there any strategies or resources the school could provide to help you manage stress better?

# a. Most important things to you about school…

1. Please can you tell me a bit about your school year – what is the best and the worst thing about being at this school over the course of the year?

**Interviews with Parents of Autistic Pupils in Y7-9 – Time 2**

**1. Educational experience prior to secondary school**

- 1. Please can you describe the kind of school your child attended prior to secondary school
  2. Can you tell me a bit about their school experience of primary school
     1. their learning
     2. support provided
     3. teacher understanding
     4. peer understanding
     5. friendships

2. **Transition to secondary school setting**

1. Can you describe the transition process from primary to secondary school
2. how you chose their secondary setting
3. the process of applying
4. support provided for your child before, during and after the transition
5. your child’s experience of the transition

**3. Diagnostic process/EHCP**

- - Can you describe your experience of the process of getting a diagnosis for your child
  - And the experience of applying for an EHCP if relevant

1. **Relationships with school staff**
2. What is your relationship with school staff like?

- are the communication channels open?
- Is the SENCO supportive?
- are subject teachers understanding?
- are staff receptive to you approaching them to inform them of your child’s needs?

1. **Child’s experience at school**

- Can you tell me what you know about your child’s experience of school
- How happy are they to go to school?
- How motivated are they by schoolwork?
- How do they get on with homework assignments?
- Do they experience any peer relationship problems?
- Are they well supported by their teachers?
- (For SRC students) What is their experience of the SRC (positives/negatives)?

1. **Impact on the family**

**a.** How has your child’s secondary school experience affected the family?

- Deciding the best school setting
- Dealing with worries/anxieties they may have had
- Meeting the needs of your other children/family members

1. **Hope and plans**
2. How do you see things going in the future for your child?
   - Academic outcomes
   - Social outcomes
   - Quality of life
3. To what extent has the school influenced these outcomes?

**Any other things you wish to add?**

**Interviews with Parents of Autistic Pupils – Time 5**

1. **Child’s experience at school**

Can you tell me what you know about your child’s experience of school

**Wellbeing**

- How happy are they to go to school?
- Has this changed at all as they’ve moved through the school?

**Learning**

- How motivated are they by schoolwork?
- Has this changed at all as they’ve moved through the school?
- How do they get on with homework assignments?

**Peer relationships**

- Do they experience any peer relationship problems?
- Has this changed at all they’ve moved through the school?

**Support from teachers**

- Are they well supported by their teachers?
- Has the level of support changed, or stayed the same?

**Challenges**

- Have they had any particular challenges over the last whole year?

**Successes**

- Have they had any successes?

**SRC (for SRC students only)**

- What is their experience of the SRC (positives/negatives)?
- Has how they use the SRC changed as they’ve transitioned through the school?

1. **Relationships with school staff**
2. What is your relationship with school staff like?

- are the communication channels open?
- Is the SENCO supportive?
- are subject teachers understanding?
- are staff receptive to you approaching them to inform them of your child’s needs?
- Have there been any changes to this over the last two year?

1. **Impact on the family**

**a.** How has your child’s secondary school experience affected the family?

- Dealing with worries/anxieties they may have had
- Meeting the needs of your other children/family members

1. **Hope and plans**
2. How do you see things going in the future for your child?
   - Academic outcomes
   - Social outcomes
   - Quality of life
3. To what extent has the school influenced these outcomes?

**Any other things you wish to add?**

**Interviews with Students – Time 5**

**1. Your experience of learning in school**

- How long have you been at this school?
- Thinking about your school in general, if you had to sell it to someone who was thinking about coming here, what positive things would you tell them?
- In contrast, what things would wish to warn them about?
- Can you tell me a bit about the lessons you like most and/or lessons you think you are good at?
- *(If pupil mentions a particular subject they like)* So what is it about [*subject]* that you like and why do you think you are good at it?
- Are there any lessons you don’t like and/or think you are not very good at, and if so, can you tell me why you don’t like them?
- Do you have any lessons away from the rest of the class? If so, how do you feel about this?
- Has experience of learning changed as you moved up the school - if so, how?

**2. Your use of school support**

- (For SRC students only) can you tell me a bit about how you use the SRC?
  - lesson times, break times, changing etc
- [Others] how much do you make use of the specialist centre/SEN room? If you use it, in what ways do you use it?
- How does the SRC/specialist centre/SEN room support you
  - Facilities, resources, staff, environment, social space etc
  - *What do you think of how your teachers treat you? (e.g. fair/understanding/respectful/reliable)*
  - *(For SRC students) Do teachers treat you differently inside and outside of the SRC? If so, how?*
- Does a teaching assistant help you in any of your lessons, and if so, in what way do they help with your learning?
- How do you feel about coming into school each day?

**3. About your friends**

- - Are you satisfied with the friendships you have at school?
  - Please can you tell me how you feel you get on with people at school?
  - If no:
- Do you have any friends outside of school? If so,
- What do you do with them?
  - Are there any friends that you had in the past who you don’t see any more? If so, what happened?

**4. Your thoughts about whether you have been treated unfairly by fellow school pupils**

- Have any pupils at school ever done or said something you didn’t like? If so, please can you tell me a bit about what happened?
- *[If they answered yes…]* Why do you think those pupils treated you that way?
- Who helped to sort out the problem, and did that problem get sorted out?

**5. About autism**

- (For SRC students) Do you tell friends in the mainstream school that you have a place in the SRC? If so, are you proud to have a place? If not, why do you choose not to tell them?
- Who in the school knows about your autism?
- Do you tell friends that you are autistic? If so, how do they respond? Do you find this helpful? If not, why do choose not to tell them?
- Do you think all of your teachers know you are autistic? If so, how does this help you? If not, do you think your school experience would be different if they did know/understand? How?
- Some people are very happy with their autism diagnosis and others are less so. How do you feel about it?

**Interviews with Staff – Time 2**

1. **Operational elements (for Head of SRC/SENCO of MSRC)**
2. Please can you outline the staffing structure [within the SRC / within the SEND dept]
3. How successful is staff retention? Have any members of the Centre/SEND Dept left or been recruited I the last academic year? Do you know the reasons for departure?
4. What if any are the practical challenges of running the SRC/SEND dept (e.g., staffing, resources, building/physical setting etc)
5. (For Heads of SRC) Funding – are any infrastructure costs met by the school? What kind of costs?
6. **Admissions (For Head of Centre)**
   1. How are admissions administered? - please can you outline the policy and practice. How successful is this process?
   2. How is the transition from Y6-Y7 administered and how successful is this process? Can you talk about the suitability of students who come to the Centre/this school – where do they come from?
   3. What is the level of attendance and have there been any placement breakdowns this year?
   4. Where do SRC/autistic students progress to?
   5. [SRC] – what has been your experience of the increased intake of autistic pupils into the MSRC as a result of the success of the SRC?
7. **Support for autistic pupils and perceived success of the centre/school**
   1. What kinds of adaptations and strategies are implemented (by the SRC/SEND dept/teachers) for autistic pupils?
   2. Are peer support interventions used, and if so, what kind and for what duration?
   3. How do autistic students (SRC/MSRC/NC) make use of the physical space (in the SRC/SEND Centre/room)? What are the most and least effective elements of the space?
   4. What are your perceptions of the success of the SRC/school in meeting the needs of autistic pupils and of inclusion in general?
   5. Are there any particular strategies that have been used to improve the school ethos (e.g. to increase autism acceptance and understanding)?
8. **Staff training and self-efficacy**
   1. What kind of autism training is provided and received for SRC staff / SEND team staff / whole school staff (teachers/auxiliary staff) – what would you say is the average number of hours staff receive?
   2. What is your own experience of autism training and how useful was it?
   3. How confident are you, and the staff (SRC/SEND/whole school) in dealing with the needs of autistic students, and in addition, with mental health issues that arise? Can you give any examples of this?
   4. Do SRC staff/SEND staff pool their knowledge/experience with staff within and/or outside of their school? What is the impact of this?
9. **Relationships**
   1. Can you talk a bit about the kinds of relationships you have with autistic pupils and their parents? (e.g., is communication open/positive or more challenging; can you give some examples?)
   2. What kind of relationship do you have with the local authority? Are there any challenges/areas for improvement?
   3. [SRC] What kind of relationship do you have with the NAS? Are there any challenges/ideas for improvement?

**Focus Group with Heads of SRC – Time 5**

**Part one: Operational elements**

**1) Staffing structure:** Please can you outline the staffing structure here within the SRC and any changes that have taken place in the last two years?

**2) Staff retention:** Have you had any members of the Centre leave in the past two years, or any new members recruited?

**3) Operational challenges:** Can you outline any practical challenges of running the Centre (*Prompts: thinking about staffing, resources or the physical space itself)*

**4) Accreditation:** Please can you describe your experience of the accreditation process

- How useful was the training you received as part of the accreditation?
- Did the accreditation impact practices within the SRC or wider school (If so, how?)
- Do you, or did you, make use of the MyProgress Framework? If so, how did you find this?

**Part two: SRC Students**

**1) Admissions:** Can you outline the policy and practice for admissions?

**-** Do you feel it is effective?

**-** Has anything changed in the admissions policy in the last two years?

**2) Integration:** What is the average level of integration for SRC students within the mainstream (*greater or less than 80/20*)?

**3) Transitions:** What has the transition been like for year 6 students moving into year 7?

- How suitable have students been that have been allocated spaces in the SRC?
- How does this compare to previous years?

**4) Attendance and placement breakdowns:** How has attendance generally been for SRC pupils this year?

- Have there been any placement breakdowns this year? If so, what prompted this?
- How do both compare to previous years?
- What does a pupil who will benefit from a SRC placement look like? *(Do you feel there are certain characteristics in pupils that suit this model better than others?)*

**5) Relationship with students:** How are relationships between staff and pupils in the Centre?

- How does this compare to the relationship with staff in the mainstream school?

**Part three: Resourcing and physical space**

**1) Use of space:**

- How do SRC students generally make use of the physical space?
- What do you feel are the most and least effective elements of the space?
- Have you made any adjustments or changes to the physical space in the last two years?

**Part four: The wider school community**

**1) Non-SRC Staff:**

- Do you feel that autism understanding amongst staff in the school has changed or remained the same over the last two years?
- How confidence would you say that staff across the school are in dealing with mental health, autism and anxiety?
- What kinds of training do staff receive?

**2) Non-autistic peers:**

- What is your perception of how the wider student populations view the SRC?
- What methods do you use to increase acceptance and understanding?

**3) Pooling of knowledge:** How is information about autism and autistic students shared across staff in the school?

- Has any CPD been offered?
- Has pooling of knowledge changed at all over the last two years?

**5) School Ethos:** How would you describe the overall school ethos towards neurodiversity?

- How integrated does the SRC feel within the school?
- Has the school ethos changed at all over the last two years (*if so, how?)*
- What is school leaderships approach to meeting the needs of autistic students?
- How involved are school leadership with the SRC?

**Part five: External relationships**

**1) Home-school communication**

- What level of communication do you typically have with the parents of SRC students?
- What channels of communication exist?

2) Please can you describe the relationship and level of communication you have with each of the following groups (e.g. how often you communicate with them and about what matters):

- With NAS
- With the Local Authority
- With other SRCs
